# Dynamic coactivation patterns during repetitive negative thinking: A cross-sectional fMRI study

**DOI:** 10.1017/S0033291726103572

**Published:** 2026-03-05

**Authors:** Marvin Sören Meiering, Emily Belleau, David Weigner, Rebecca Gruzman, Diego Pizzagalli, Sören Enge, Simone Grimm

**Affiliations:** 1 Institute of Neuroscience and Biopsychology for Clinical Application, MSB Medical School Berlin, Rüdesheimer Straße 50, 14197, Berlin, Germany; 2 Department of Education and Psychology, Freie Universität Berlin, Habelschwerdter Allee 45, 14195, Berlin, Germany; 3 Center for Depression, Anxiety and Stress Research, McLean Hospital, Belmont, MA, USA; 4 Department of Psychiatry, Harvard Medical School, Boston, MA, USA; 5 Department of Psychiatry, Psychotherapy and Psychosomatics, Psychiatric University Hospital Zurich, University of Zurich, Zurich, Switzerland

**Keywords:** default mode network, frontoparietal network, fMRI, neuroticism, repetitive negative thinking, salience network

## Abstract

**Background:**

Repetitive negative thinking (RNT) and neuroticism are risk factors for internalizing psychopathology. However, their interaction has only been investigated at the self-report level, and studies elucidating their interrelationship at the neural level are lacking. We therefore investigated the interaction of trait RNT and neuroticism with respect to the dynamics of neural networks during negative self-referential processing.

**Methods:**

A sample of 110 healthy subjects reported trait RNT and neuroticism, followed by an RNT induction paradigm during fMRI. Dynamic coactivation pattern (CAP) analysis was used to identify a set of recurring coactivation patterns and to quantify their persistence and count rates. Next, the effects of trait RNT, neuroticism, and their interaction on brain dynamics were tested using regression models.

**Results:**

Negative interactions between RNT and neuroticism were found for persistence and counts of the canonical default mode network (DMN) as well as salience network (SAL) CAP. Simple slope analysis revealed that subjects scoring high on neuroticism exhibited a negative association between trait RNT and DMN as well as canonical SAL dynamics. Furthermore, trait RNT was positively associated with persistence and count rates of a hybrid FPN+DMN coactivation state.

**Conclusions:**

Our results suggest that individuals with high neuroticism who spend more time in SAL and DMN CAPs may be less vulnerable to RNT, potentially reflecting more adaptive network configurations. Furthermore, less segregated CAPs, evident by the concurrent activation of functionally antagonistic networks (FPN+DMN), emerge more often in individuals prone to RNT, likely reflecting disrupted network interactions.

## Introduction

Repetitive negative thinking (RNT) is defined as the persistent or intrusive self-referential thinking about past or potential future negative experiences, and is particularly relevant to internalizing disorders (Ehring, 2021; Ehring & Watkins, 2008). Although primarily studied in clinical populations, RNT is also a dimensional trait found in healthy individuals, where it increases vulnerability to psychopathology, as evidenced by its association with the onset and persistence of mood disorders (Ehring & Watkins, 2008; Hijne, Penninx, Van Hemert, & Spinhoven, 2020; Samtani et al., 2022; Spinhoven, Van Hemert, & Penninx, 2018; Taylor & Snyder, 2021; Zagaria, Ballesio, Vacca, & Lombardo, 2023). Investigating the neural correlates of RNT may improve our understanding how internalizing disorders are represented in the human brain. Eventually, leading to the discovery of targetable biomarkers and improving the treatment of patients affected by the adverse consequences of RNT. In the past, RNT has been most commonly investigated in its two disorder-specific facets: rumination and worry (Moulds & McEvoy, 2025). Rumination is characterized by a past-oriented thinking mode associated with depression, whereas worry is typically future-oriented and linked to anxiety disorders (Ehring & Watkins, 2008; Moulds & McEvoy, 2025). Both are centrally defined by excessive self-referential thinking as well as disengagement difficulties and have been repeatedly shown to load on their common factor RNT (Ehring & Watkins, 2008; Moulds & McEvoy, 2025). In the following, we will use RNT as an umbrella term spanning both, rumination and worry.

Alterations of the default mode network (DMN), salience network (SAL), and frontoparietal network (FPN) – collectively termed the triple-network model – have been linked to both normative and atypical mental states (Menon, 2011, 2023). The DMN is associated with internal self-referential processing and deactivates in response to cognitively demanding mental processes (Greicius, Krasnow, Reiss, & Menon, 2003; Raichle et al., 2001). In contrast, the FPN is implicated in cognitively demanding processes like attentional control (Menon, 2011; Menon & D’Esposito, 2022; Seeley et al., 2007). The SAL, also referred to as the ventral attention network, is primarily responsible for the detection of salient stimuli and the reallocation of mental resources (Menon, 2011, 2023; Seeley, 2019; Seeley et al., 2007; Sridharan, Levitin, & Menon, 2008; Uddin, 2015). In mental disorders, the dynamic interactions between the DMN, FPN, and SAL can derail, resulting in prolonged network recruitment or deactivation when external demands would require inter-network flexibility and organized coordination (Menon, 2011). A wealth of evidence suggests that this inability to flexibly switch between large-scale networks in response to environmental demands may underlie the debilitating repetitiveness and excessive internal focus that characterizes RNT (Belleau, Taubitz, & Larson, 2015; Hamilton et al., 2011; Kaiser, Andrews-Hanna, Wager, & Pizzagalli, 2015; Koster, De Lissnyder, & De Raedt, 2013; Koster, De Lissnyder, Derakshan, & De Raedt, 2011; Lydon-Staley et al., 2019).

Although extensively studied using static neuroimaging methods (Makovac, Fagioli, Rae, Critchley, & Ottaviani, 2020; Meiering, Weigner, Gruzman, Enge, & Grimm, 2025), recent evidence links the non-stationary nature of brain network interactions to RNT (Belleau et al., 2022; X. Chen et al., 2020; Kaiser et al., 2019; Peterson, Smolker, Moser, & Kaiser, 2025). In this context, dynamic coactivation pattern (CAP) analysis has emerged as a promising method (Bolton et al., 2020; J. E. Chen, Chang, Greicius, & Glover, 2015; X. Liu, Chang, & Duyn, 2013). With CAP, a recurrent set of brain patterns characterized by concurrent (de)activation is identified and analyzed on the level of individual volumes. In contrast to traditional dynamic functional connectivity approaches, which rely on arbitrarily defined sliding windows (Allen et al., 2014), CAP analysis captures time-resolved network configurations without imposing temporal windowing. Herein, rumination has been linked to an increased time spent in a hybrid CAP that simultaneously engages the FPN, DMN, and SAL at rest (Belleau et al., 2022; Kaiser et al., 2019). Moreover, increased transitions between this hybrid brain state and the canonical DMN were found to relate to rumination (Belleau et al., 2022; Kaiser et al., 2019; C. Liu et al., 2023). These findings suggest that individuals with high trait RNT may struggle to coordinate the recruitment of circuits related to salience processing (SAL), cognitive control (FPN), and self-referential processing (DMN). Aberrant time-dependent coordination of these brain networks likely underlies difficulties in disengaging from negative self-referential thinking, potentially preventing the generation of adaptive behavioral responses (Koster et al., 2011; Martin & Tesser, 1989, 1996; Watkins, 2008). Surprisingly, dynamics of the canonical DMN itself were inconsistently linked to RNT facets (Belleau et al., 2022; Kaiser et al., 2019; C. Liu et al., 2023; Piguet, Karahanoğlu, Saccaro, Van De Ville, & Vuilleumier, 2021). To our current knowledge, RNT has been exclusively studied during the resting state. However, no study has yet examined the relationship between RNT and the dynamics of large-scale networks during experimentally induced negative self-referential processing. Accordingly, we hypothesize a positive association between the occurrence rate of (hybrid) DMN configurations and self-reported RNT during experimentally induced RNT. Moreover, this association is hypothesized to be driven by the persistence of the CAP configurations.

RNT is critically dependent on negative mood — whether transient, as seen in typical emotional responses to adverse experiences, or persistent, as commonly observed in individuals with high neuroticism (Marchetti, Koster, Klinger, & Alloy, 2016; Watkins, 2008; Whitmer & Gotlib, 2013). Traditionally, neuroticism is defined as the tendency to experience negative affect, emotional instability, and heightened reactivity to aversive stimuli (DeYoung, 2015; Eysenck, 1967). Moreover, neuroticism is considered a non-specific vulnerability marker for psychopathology, as it predicts common mental disorders but has limited ability to distinguish between etiologically distinct subgroups (Ormel et al., 2013; Ormel, Rosmalen, & Farmer, 2004). A substantial body of evidence points to a multifaceted relationship between neuroticism and RNT (Iqbal & Dar, 2015; Merino, Ferreiro, & Senra, 2014; Merino, Senra, & Ferreiro, 2016; Zvolensky et al., 2016). Herein, Watkins (2008) and Marchetti et al. (2016) proposed an amplification hypothesis, stating that repetitive/spontaneous thought processes interact bidirectionally with negative affect (at both trait and state levels), thereby increasing vulnerability to psychopathology. Building on these prior conceptualizations, our group has specifically focused on interactions between RNT and neuroticism (Meiering et al., 2025; Meiering, Weigner, Enge, & Grimm, 2023), proposing that high levels of neuroticism amplify the detrimental impact of RNT facets. As a consequence, the combined effect of RNT and neuroticism is expected to confer psychopathological risk beyond either factor alone. Recent studies using static neuroimaging methods have demonstrated that the DMN – central to RNT – is also associated with neuroticism (Aghajani et al., 2014; Chou, Deckersbach, Dougherty, & Hooley, 2023; Gentili et al., 2017; Zhi et al., 2024). This convergence positions the DMN as a promising neural target for investigating RNT-by-neuroticism interactions. Accordingly, we hypothesize that a positive interaction of trait RNT and neuroticism is reflected by increased occurrence rates of the DMN. Moreover, we predict that this interaction is driven by increased persistence of the DMN. In exploratory analyses, the same analytical approach is applied to other brain configurations involving networks represented in the triple network model (Menon, 2011).

## Methods

**Figure 1.**
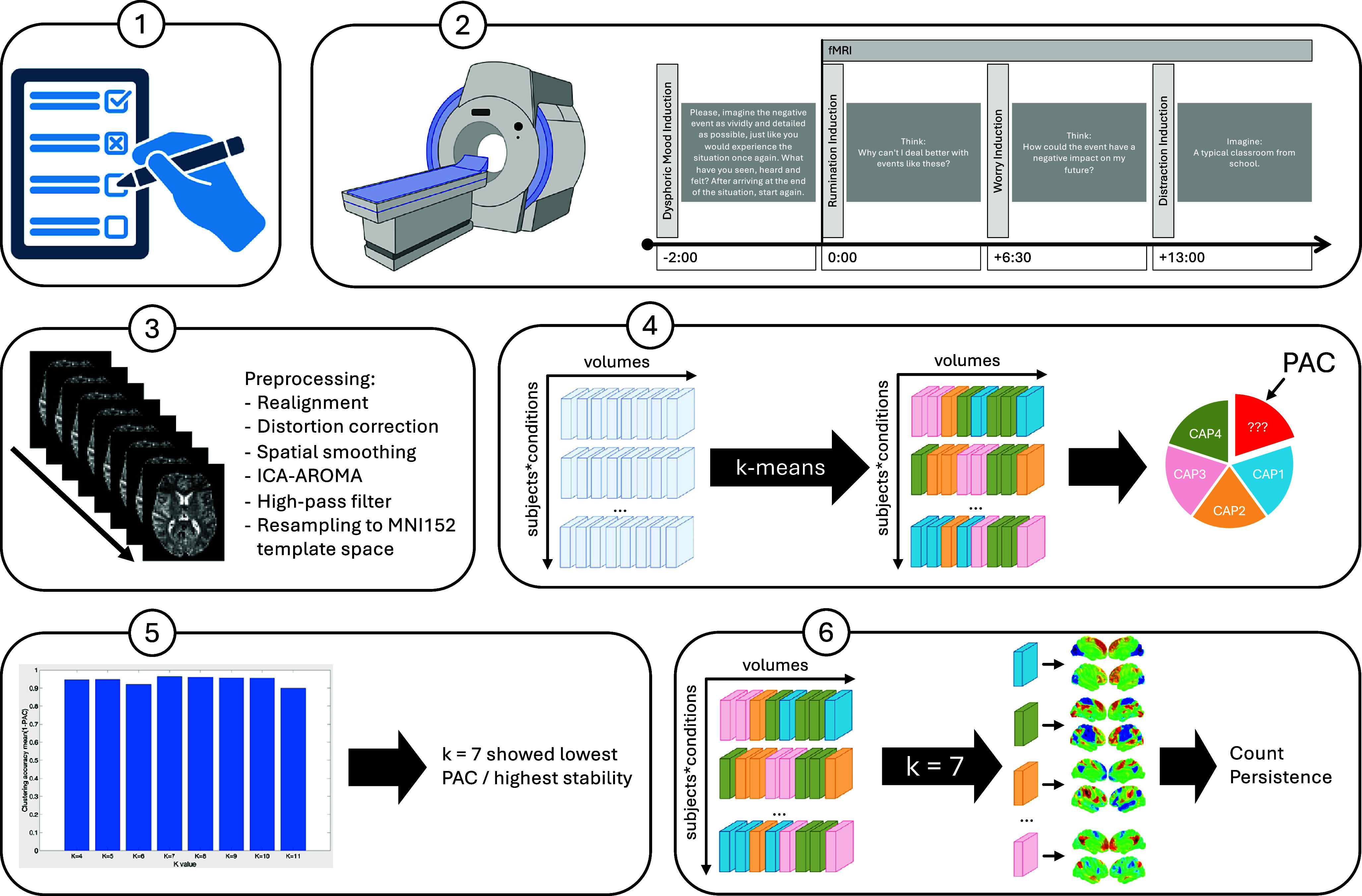
Analytical pipeline. *Note*: 1) Several self-report questionnaires were obtained probing facets of RNT and neuroticism. 2) Then, subjects underwent a RNT induction task during fMRI. 3) The fMRI data were preprocessed and resampled to the MNI152 template space. 4) K-means clustering was performed 50 times using a random subsample comprising 80% of the volumes from all subjects to cluster volumes into a finite set of recurring coactivation patterns. Proportion of ambiguously clustered pairs was calculated to determine the stability of the clustering solutions. The process was repeated for k = 4 to k = 11 to conclude a winning parameter. 5) PAC was compared across the different k-means iterations to determine the clustering solution with the greatest stability. K = 7 turned out to be the winning parameter with a mean stability of 96.5%, performing nominally better than all other clustering solutions that were tested. 6) Finally, k-means clustering was performed again – using all volumes from all subjects – to determine the spatial layout of the final CAPs, their counts/occurrences and persistence. The figure was in part created with BioRender.

A detailed protocol outlining all paradigms, questionnaires, hypotheses, procedures, and analytical approaches used in this study has been previously published by our group (Meiering et al., 2023). An overview of the analytical pipeline is given in Figure 1.

### Participants

Only healthy adults between 18 and 45 years were enrolled. Exclusion criteria included receiving a diagnosis for a mental disorder, psychotherapy or psychopharmacological treatment in the past 12 months, pregnancy, or meeting any of the MRI exclusion criteria. Furthermore, subjects were instructed to refrain from alcohol and other psychoactive substances 48 h before the MRI assessment. Subjects were recruited using e-mail, personal approach, and advertising of the study in the online management system for psychological studies conducted at MSB Medical School Berlin and Freie Universität Berlin. The study was approved by the institutional review board of the MSB Medical School Berlin, and all subjects provided written informed consent to participate.

### Procedure

After determining participants’ eligibility through an online screening, several self-report questionnaires were utilized to assess trait constructs (detailed below). Next, participants underwent functional magnetic Resonance Imaging (fMRI) while completing a Rumination and Worry Induction Task (RWIT; based on (X. Chen et al., 2020; Cooney, Joormann, Eugène, Dennis, & Gotlib, 2010; Kowalski, Wypych, Marchewka, & Dragan, 2019; Nolen-Hoeksema & Morrow, 1993; Paulesu et al., 2010) to investigate neural changes associated with RNT.

Given recent evidence suggesting that RNT is contingent on negative mood (X. Chen et al., 2020; Whitmer & Gotlib, 2013), participants underwent a dysphoric mood induction procedure inside the scanner, immediately before the RWIT started. This involved recalling an autobiographical event that elicited negative emotions such as guilt, shame, sadness, or fear, and vividly imagining this experience for 2 minutes. Next, participants were instructed to reflect on the negative event using self-oriented questions designed to elicit either rumination (RUM) or worry (WOR). For example, a question intended to evoke rumination was ‘Why can’t I handle events like this better?’, while a worry-inducing question was ‘How can the event affect my future negatively?’ (all stimuli are given in the Supplement). As a contrast condition, participants were presented with four brief neutral sentences describing everyday situations – such as ‘A typical classroom from school’ – and asked to vividly imagine them (distraction; DIS). Each condition comprised four prompts (questions or sentences), displayed for 90 seconds, interspersed by a 30-second instruction slide. The rumination and worry conditions were presented in a randomized order, whereas the distraction condition was always presented last to ensure that rumination and worry were undergone continuously. In total, completion of the RWIT took approximately 22 min. At the analytical stage, the RUM and WOR conditions were averaged to obtain neural measures reflecting their common denominator, RNT. All hypotheses were tested twice using data from the RNT condition and RNT > DIS contrast.

### Psychometric assessments

Following recent suggestions to increase the reliability of self-reports (DeYoung et al., 2025), a confirmatory factor analysis (CFA) was employed to obtain factor scores of RNT and neuroticism. In this context, the information provided by several questionnaires probing overlapping constructs are aggregated into factor scores by removing variance that is not attributable to their common latent factor, thereby enhancing the validity and sensitivity of statistical analyses (DiStefano, Zhu, & Mîndrilã, 2009).

To aggregate a reliable measure of RNT, the CFA included the Brooding subscale from the Response Styles Questionnaire (Huffziger & Kühner, 2012; Treynor, Gonzalez, & Nolen-Hoeksema, 2003), the Penn State Worry Questionnaire total score (Meyer, Miller, Metzger, & Borkovec, 1990; Stöber, 1995), the rumination subscale of the Cognitive Emotion Regulation Questionnaire (Loch, Hiller, & Witthöft, 2011), and the Perseverative Thinking Questionnaire total score (Ehring et al., 2011).

A similar approach was used to assess neuroticism. The Negative Affect subscale of the trait Positive and Negative Affect Schedule (Breyer & Bluemke, 2016; Watson, Clark, & Tellegen, 1988), the Negative Affectivity scale of the Adult Temperament Questionnaire (Rothbart, Ahadi, & Evans, 2000; Wiltink, Vogelsang, & Beutel, 2006), the Negative Emotionality subscale from the Big Five Inventory 2 and Neuroticism from the Big Five Inventory short version (D. Danner et al., 2016; Daniel Danner et al., 2019; Rammstedt & John, 2005; Soto & John, 2017) as well as the trait Anxiety subscale from the State Trait Anxiety Inventory (Bertrams & Englert, 2013; Spielberger, Gorsuch, Lushene, Vagg, & Jacobs, 1983) were aggregated into a factor score using CFA. The results of the factor analyses were already published elsewhere (Meiering et al., 2025) and can be found in the Supplement.

To ensure data quality of the self-report measurements, three items were randomly interspersed into the questionnaires to assess the participants’ attention (e.g. ‘To show that you have read this question, please select “rarely applicable.”’). Only individuals who answered at least two of these items correctly were included in the analyses (DeSimone, Harms, & DeSimone, 2015). Univariate statistics of the questionnaires can be obtained from Supplementary Table S1.

### MRI acquisition and analysis

Details of the employed MRI sequences and brain image preprocessing can be found in the Supplemental Material. Briefly, the fMRI data were realigned, smoothed, denoised using ICA-AROMA (Pruim, Mennes, Buitelaar, & Beckmann, 2015; Pruim et al., 2015), detrended, and resampled to the MNI152 template space. To account for the different conditions of the RWIT paradigm, the conditions were extracted from the fMRI time series, resulting in three blocks per subject (RUM, WOR, and DIS). Then, the blocks were aliased as different subjects and included in the CAP analysis as described below. This approach enabled us to determine the number and spatial layout of recurrent CAPs based on all three conditions, thereby ensuring direct comparability between conditions. Moreover, this approach also enabled the computation of dynamic CAP features separately for each condition.

### Coactivation pattern analysis

We employed a whole-brain, seed-free, voxel-wise dynamic coactivation pattern (CAP) analysis using the TbCAPs Toolbox (Bolton et al., 2020). The seed-free variant of CAP used here has the advantage that all fMRI volumes are included in the process, presumably increasing the reliability of derived dynamic CAP metrics. Furthermore, the CAP analysis was restrained to gray matter voxels based on a gray matter mask of the MNI152 template. Consensus clustering was employed to empirically determine the number of CAPs present in the data. For each number of k clusters considered stable in previous studies (X. Liu et al., 2013), k-means clustering was run several times using a randomly selected subsample of the original data without replacement (Monti, Tamayo, Mesirov, & Golub, 2003; Zoller et al., 2019). During k-means clustering, each fMRI volume was assigned to one of the prespecified clusters, and the assignment was stored in a consensus matrix. Optimal clustering would result in fMRI volumes that are either consistently clustered together or separately over each iteration. Following suggestions from a previous study showing that clustering for k ≤ 4 or k > 11 results in less stable CAPs, we performed k-means clustering with k values of 4–11 (X. Liu et al., 2013). An optimal clustering solution for k = 7 was determined based on consensus clustering with 50 independent runs for each k, randomly sampling 80% of the original data, using the proportion of ambiguously clustered pairs as a cost metric (Șenbabaoğlu, Michailidis, & Li, 2014). Subsequently, k-means clustering with k = 7 was run over 100 folds to bypass local minima and instability of the resulting CAPs, resulting in whole-brain maps that characterize regions that are co(de)activated at the same time. Afterward, FSL’s spatial cross-correlation tool (Jenkinson, Beckmann, Behrens, Woolrich, & Smith, 2012; Smith et al., 2004) was used to determine the spatial similarity between the seven CAP maps and Schaefer’s cortical seven-network parcellation to determine correspondences between the CAPs found in our sample and previously defined networks (Schaefer et al., 2018). Finally, the total number of volumes spent in each CAP (count/occurrences) and the number of consecutive volumes spent in each CAP (persistence) were computed for each of the experimental conditions separately. Empirical as well as theoretical considerations of rumination and worry support that both share key characteristics and are centrally defined by the same underlying psychological process termed repetitive negative thinking (RNT; Ehring & Watkins, 2008; Moulds & McEvoy, 2025). Accordingly, CAP metrics derived from the rumination and worry conditions were averaged to obtain a contrast reflecting their common denominator RNT. Summing measures to approximate the expression of a given construct is a standard procedure in psychometrics (Sijtsma, Ellis, & Borsboom, 2024). We have chosen to calculate averages instead of raw sum scores to retain the original scale of the measures, thereby allowing direct comparisons between the RNT and DIS conditions. The differential contrast reflecting RNT in comparison to DIS was obtained by subtracting CAP measures (count/occurrences and persistence) during DIS from the RNT condition.

### Statistical analysis

All statistical analyses were conducted in R version 4.3.1 (Posit Software, 2023; R Core Team, 2023). Associations between neural measures and factor scores were estimated using Tukey’s bi-weight regression models with an MM-estimator. By reducing the weight of observations according to the size of their residuals, the Tukey’s bi-weight approach effectively reduces the influence of outliers on the estimates (Yu & Yao, 2017). Subsequent nonparametric bootstrapping with 10000 iterations was used to test the explanatory variable in question. To test the moderation hypotheses, trait RNT*neuroticism interaction terms with CAP metrics as dependent variables were estimated as described above. In case of no meaningful moderation, the interaction term was omitted from the model. As argued above, neuroticism is considered an unspecific risk factor for psychopathology and should, therefore, be accounted for when investigating RNT (Ormel et al., 2013, 2004). Hence, we always included neuroticism factor scores as a covariate of no interest in the model. Finally, a posteriori regression models were employed to test the associations of counts and persistence between CAPs of interest. Age and sex were included as a priori covariates of no interest in all models. In accordance with the severe flaws associated with the null-hypothesis significance testing framework and the p-value (Cumming, 2014; Cumming & Finch, 2005), results are interpreted given the effect sizes and associated confidence intervals. P-values are provided as a complementary statistic but are not interpreted.

## Results

A total of 122 subjects completed the fMRI assessments, 10 subjects were excluded from further analyses due to poor fMRI data quality (>4 mm absolute mean head motion or substantial artifacts assessed by visual inspection of the data). Additionally, 2 participants did not pass the quality check for self-reports (failing ≥2 out of 3 attention items evenly distributed across questionnaires). Accordingly, for brain-behavior relationships, a final sample of 110 subjects remained. The final sample consisted of 31 males (28.18%) and 79 females (71.82%) with a mean age of 23.42 (*SD* = 4.03) ranging from 18 to 40 years. Since recruitment primarily targeted university students, 82% of the subjects (n = 90) either already held an academic degree or were enrolled for one at the time of participation.

### Coactivation patterns

**Figure 2.**
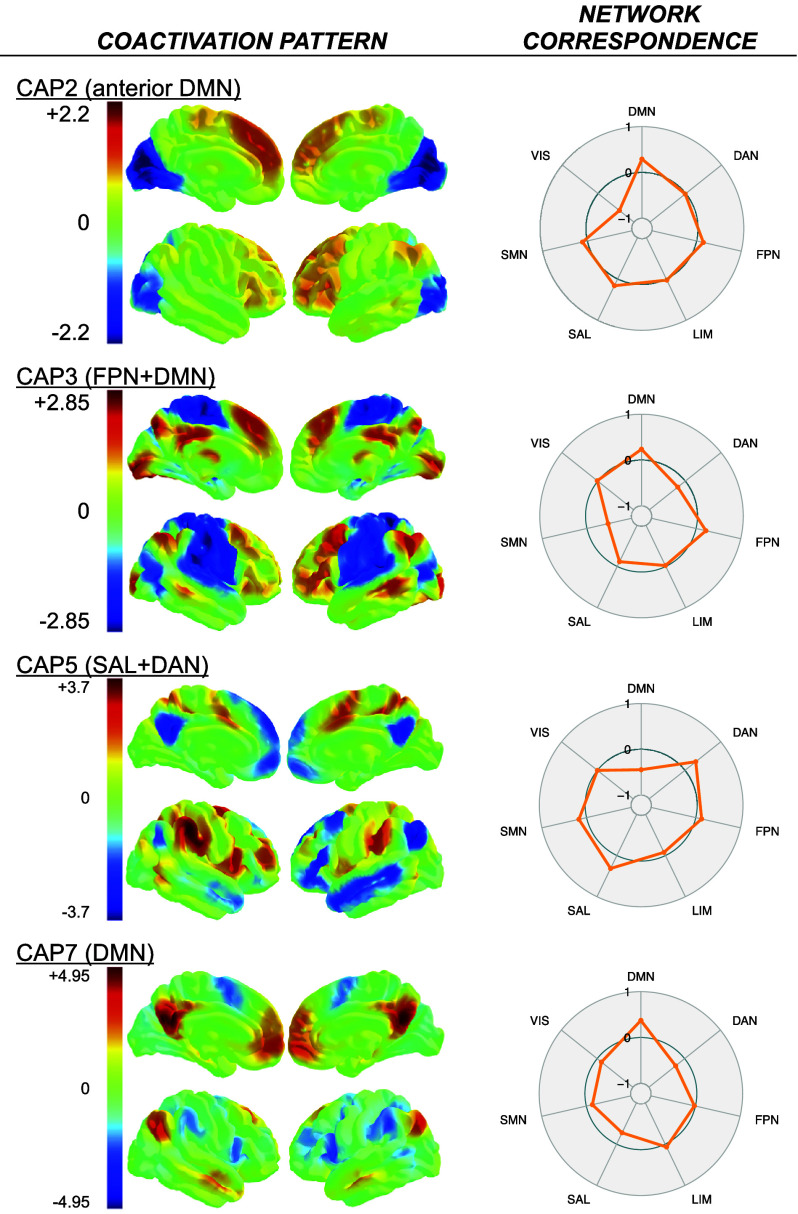
CAP maps and their corresponding spatial similarities with Schaefer’s cortical seven network parcellation. *Note*: One represents a perfect correlation of the respective network from Schaefer’s network with positive coactivations in the CAP map, whereas negative one represents perfect correlations with codeactivations in the CAP map. VIS = visual network, DMN = default mode network, FPN = frontoparietal network, SMN = sensorymotor network, SAL = salience network/ventral attention network, DAN = dorsal attention network, LIM = limbic network. Color bars reflect local z statistics of the respective CAP.

Consensus clustering identified 7 CAPs repeatedly present in the data. Spatial cross-correlation of z-transformed whole-brain CAP maps with Schaefer’s cortical 7 network parcellation (Schaefer et al., 2018) suggested CAP1 to be most strongly associated with the visual network (*ρ* = .678) and CAP2 with the anterior DMN (aDMN; *ρ* = .291, see Figure 2). CAP3 reflected a hybrid network comprising concurrent activation of the FPN as well as the DMN (FPN+DMN; FPN: *ρ* = .221, DMN: *ρ* = .236). CAP4 was associated with the somatomotor network (*ρ* = .609). CAP5 was linked to the spatial layout of the salience and dorsal attention network (VAN: *ρ* = .315, DAN: *ρ* = .302), whereas CAP7 was uniquely associated with the DMN in its canonical form (*ρ* = .373). CAP6 exhibited activation patterns located close to the edges of the brain, the ventricles, and white matter tracts (see Supplementary Figure S3), as commonly observed in images that are corrupted by head motion or susceptibility artifacts (Bijsterbosch, Smith, & Beckmann, 2017; Poldrack, Mumford, & Nichols, 2011; Salimi-Khorshidi et al., 2014). Consequently, CAP6 was discarded from further analysis. Moreover, CAP1 and CAP4 were excluded from further analyses since both reflect networks that are predominantly associated with sensory information processing and have not been implicated in the triple network model (Menon, 2011, 2023). All spatial cross-correlations can be obtained from Supplementary Table S3 and Supplementary Figure S4.

#### Task engagement

The effects of task conditions on brain dynamics are summarized in the Supplement.

#### Regression analyses

**Figure 3.**
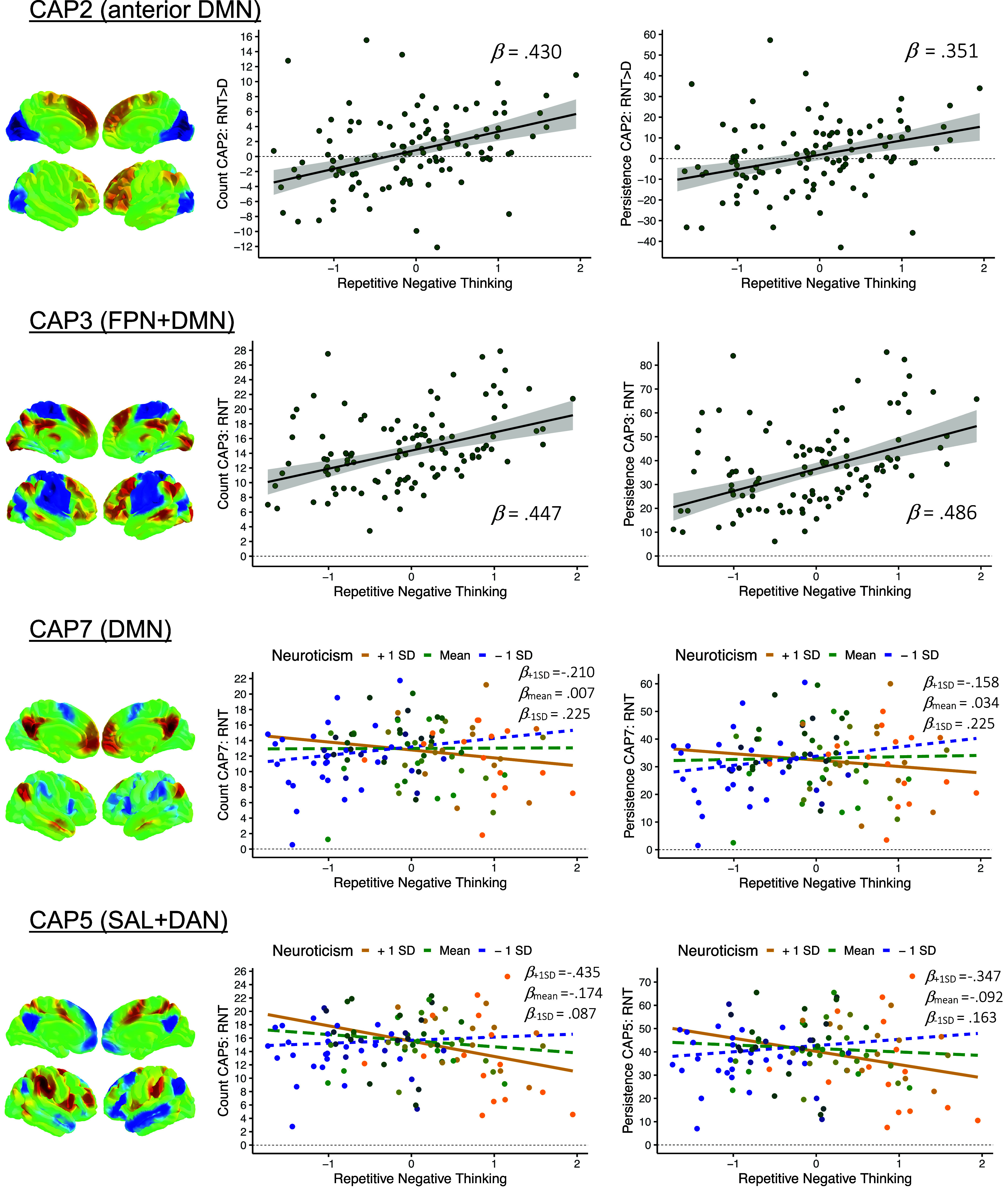
Regression results. *Note:* DMN = default mode network, SAL = salience network, DAN = dorsal attention network, FPN = frontoparietal network, RNT = repetitive negative thinking, DIS = distraction. Color bars reflect local z statistics of the respective CAP. Color coding of the data points in the moderation plots reflect expressions of neuroticism with respect to the sample.

**Figure 4.**
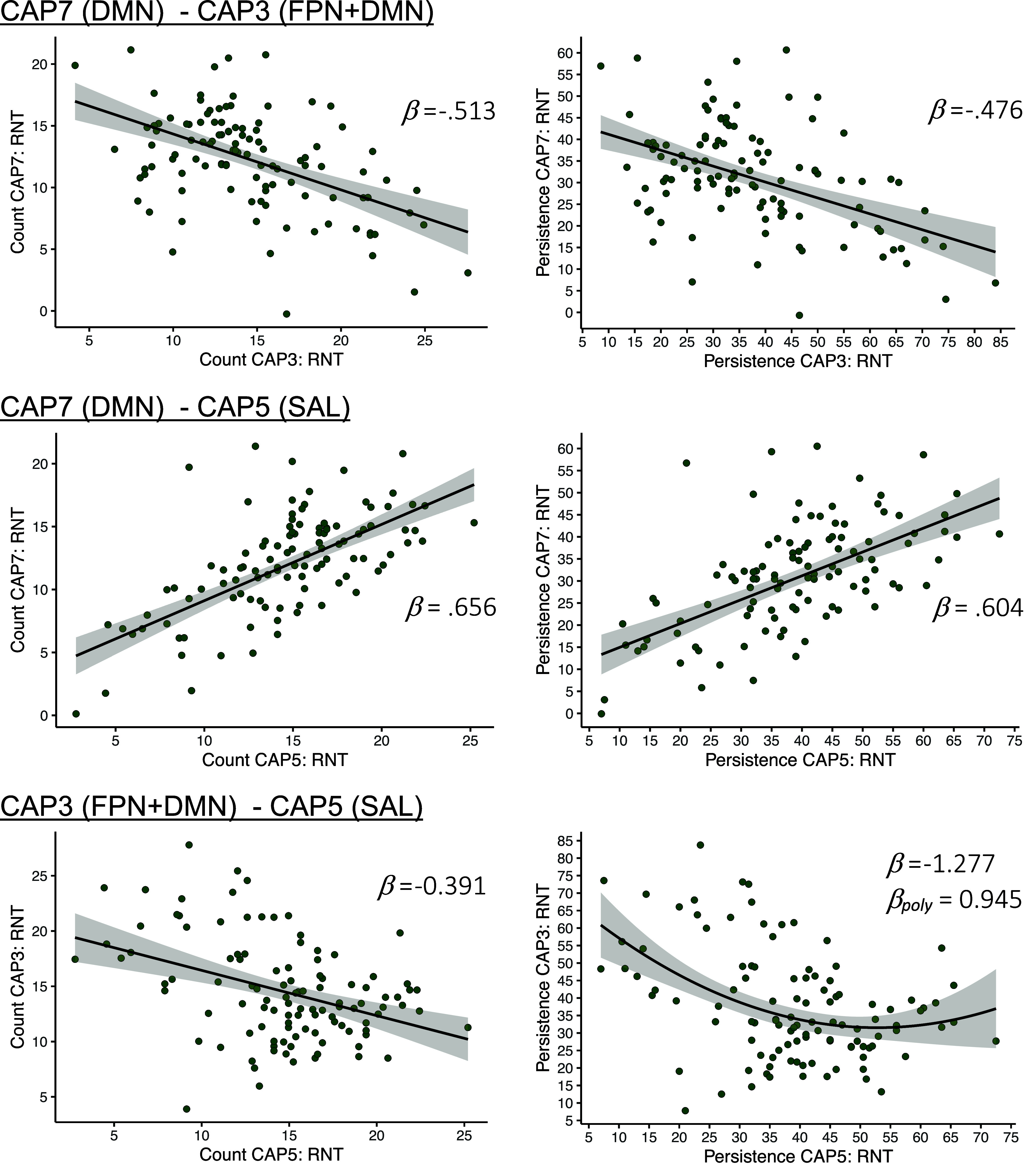
CAP-to-CAP associations. *Note:* DMN = default mode network, SAL = salience network, DAN = dorsal attention network, FPN = frontoparietal network, RNT = repetitive negative thinking.

Statistical results are summarized in Table 1 as well as in Figures 3 and 4. All statistical results can be obtained from Supplementary Table S6.

**Table 1.** Regression

CAP (contrast)	*Β*	95%-CI (Β)	β	95%-CI (β)	p	p-FDR
Trait RNT*neuroticism interaction						
Count						
CAP5 (RNT)	−2.030	[−3.694, −0.044]	−0.261	[−0.475, −0.013]	.047	.188
CAP7 (RNT)	−1.562	[−2.974, −0.173]	−0.217	[−0.414, −0.024]	.032	.164
Persistence						
CAP5 (RNT)	−6.162	[−11.152, −0.640]	−0.255	[−0.457, −0.031]	.034	.188
CAP7 (RNT)	−4.134	[−8.175, −0.226]	−0.191	[−0.378, −0.008]	.041	.164
*Trait RNT factor scores*						
Count						
CAP2 (RNT > DIS)	2.465	[0.899, 4.045]	0.430	[0.153, 0.716]	.004	.032
CAP3 (RNT)	2.468	[0.522, 4.872]	0.447	[0.085, 0.881]	.014	.056
Persistence						
CAP2 (RNT > DIS)	6.927	[0.589, 13.293]	0.351	[0.023, 0.673]	.033	.132
CAP3 (RNT)	9.233	[2.120, 17.580]	0.486	[0.106, 0.914]	.012	.056
*CAP to CAP associations*						
Count						
CAP7-CAP3 (RNT)	−0.453	[−0.614, −0.286]	−0.513	[−0.696, −0.326]	.0001	.0002
CAP7-CAP5 (RNT)	0.607	[0.486, 0.731]	0.656	[0.524, 0.796]	.0001	.0002
CAP3-CAP5 (RNT)	−0.410	[−0.590, −0.234]	−0.391	[−0.561, −0.225]	.0004	.0005
Persistence						
CAP7-CAP3 (RNT)	−0.367	[−0.491, −0.233]	−0.476	[−0.635, −0.300]	.0001	.0002
CAP7-CAP5 (RNT)	0.540	[0.415, 0.662]	0.604	[0.468, 0.739]	.0001	.0002
CAP3-CAP5 (RNT)	−1.481	[−2.443, −0.776]	−1.277	[−2.094, −0.659]	.0006	.0006

*Note:* Β = unstandardized coefficient, β = standardized coefficient, CI = bootstrap confidence intervals, DIS = distraction, RNT = repetitive negative thinking, CAP2 = aDMN, CAP3 = FPN+DMN, CAP5 = SAL+DAN, CAP7 = DMN. All results are corrected for the effects of age and sex. Associations including RNT factor scores were additionally corrected for neuroticism.

CAP2 (aDMN) counts were positively related to RNT factor scores in the RNT > DIS contrast, but not during RNT alone. Confidence intervals indicated a small to large effect size that is most likely to be positive. The same applied for persistence of CAP2 (aDMN). Conversely, CAP3 (FPN+DMN) counts were moderately associated with RNT factor scores during RNT, but not for the RNT > DIS contrast. The confidence interval indicated that the effect is most likely to be positive, although associated with some degree of variability. Visual inspection of the scatter plot for CAP3 suggested a quadratic relationship, so model comparison was conducted to determine whether the inclusion of a polynomial term improves model fit. Model comparison failed to provide evidence for superior explanatory power of the model including the polynomial term (*ΔR^2^* = 3.491%, *95%-CI* = [−5.414, 12.427], *p_perm_* = .149, *ΔBIC_poly-null_* = 0.871). Accordingly, the polynomial term was omitted from the model. Similarly, a quadratic relationship was inferred from visual inspection of the scatter plot for CAP3 persistence and trait RNT. The results from model comparisons including the polynomial term were inconclusive (*ΔR^2^* = 4.176%, *95%-CI* = [−4.219, 14.383], *p_perm_* = .038, *ΔBIC_poly-null_* = 0.046). While bootstrap confidence intervals and Bayesian Information Criterion suggested no improved explanatory power, the permutation-based p-value indicated otherwise. Eventually, the polynomial term was omitted from the model, since the polynomial term itself was not significant (*B* = 3.118, *95%-CI* = [−1.004, 8.040], *β* = .157, *p* = .131).

A negative RNT*neuroticism interaction emerged for CAP7 (DMN) counts and persistence during RNT. Confidence intervals suggest that the effect is likely to be small to moderate. Exploratory analysis of the SAL CAP (CAP5) revealed a negative RNT*neuroticism interaction during RNT. Confidence intervals indicated that the interaction effects are most likely negative, although associated with considerable variability. RNT*neuroticism interactions for CAP2 (aDMN) and CAP3 (FPN+DMN) dynamics showed marginal effect sizes associated with inconclusive confidence intervals. For the RNT > DIS contrast, no meaningful trait RNT*neuroticism interactions were found.

A posteriori analyses revealed several meaningful associations between CAP3, CAP5, and CAP7 during RNT, for counts as well as persistence (see Table 1 and Figure 4). Specifically, CAP3 (FPN+DMN) and CAP7 (DMN) were negatively associated with each other, with confidence intervals strongly indicating a moderate to large effect. For CAP5 (SAL) and CAP7 (DMN), a positive relationship was found, associated with narrow confidence intervals suggesting the effect to be most likely of large magnitude. A negative association with confidence intervals indicating an effect of small to large magnitude was found for counts of CAP3 (FPN+DMN) and CAP5 (SAL). Similarly, a negative association was found for the association between CAP3 and CAP5 persistence. Notably, visual inspection of scatter plots suggested a nonlinear pattern for the association between CAP3 (FPN+DMN) and CAP5 (SAL) for counts as well as persistence. Accordingly, a polynomial term was added to the models, and model comparisons were conducted. For counts, confidence intervals of the determination coefficient, the p-value as well as Bayesian Information Criterion indicated no improved explanatory power of the model including the polynomial term (*ΔR^2^* = 3.380%, *95%-CI* = [−0.444, 11.749], *p_perm_* = .080, *ΔBIC_poly-null_* = 1.003). Accordingly, the polynomial term was omitted from the model. For the association between CAP3 and CAP5 persistence, model comparison results were inconclusive (*ΔR^2^* = 4.568%, *95%-CI* = [−1.004, 8.193], *p_perm_* = .039, *ΔBIC_poly-null_* = −0.400). While the confidence intervals indicated considerable variation of the determination coefficient, the permutation-based p-value as well as the Bayesian Information Criterion indicated improved explanatory power of the polynomial model. Eventually, the polynomial term was kept in the model because the polynomial term itself showed a large effect size associated with confidence intervals indicating a positive effect to be most likely (*B* = 0.014, *95%-CI* = [0.005, 0.026], *β* = 0.945, *p* = .004).

## Discussion

The present study employed dynamic CAP analysis to investigate the neural correlates of RNT by neuroticism interactions in a cross-sectional sample of healthy adults. Our findings demonstrate that persistence and counts of the anterior DMN and hybrid FPN+DMN CAPs exhibited strong positive associations with trait RNT, irrespective of neuroticism levels. Additionally, a modulating effect of neuroticism on the link between trait RNT and time spent in the SAL and DMN CAPs was found. Finally, the hybrid FPN+DMN CAP showed negative associations with both the SAL and canonical DMN CAPs, while a positive association was observed between the SAL and DMN CAP.

Consistent with our hypothesis, a positive association was found between trait RNT and counts of the anterior DMN CAP during RNT compared to distraction. In the past, static as well as dynamic features of the anterior DMN have been associated with RNT and depression (Kim et al., 2023; Makovac et al., 2020; Sheline, Price, Yan, & Mintun, 2010). Speculatively, the persistence of an anterior DMN CAP during negative self-referential processing might reflect excessive self-referential thinking characterizing RNT (Ehring, 2021; Ehring & Watkins, 2008). Additionally supporting this notion, we also observed a positive relationship between trait RNT and occurrence rates of a hybrid FPN+DMN CAP. The hybrid FPN+DMN CAP suggests concurrent activation of functionally antagonistic networks during negative self-referential processing. We cautiously speculate that this result might indicate an inability to effectively coordinate network recruitment in individuals prone to RNT. The association is driven by the persistence of the hybrid FPN+DMN network configuration. Together, these findings support the hypothesis that (prolonged) time spent in coactivation configurations comprising functionally antagonistic brain networks might play a key role for trait RNT (Belleau et al., 2022, 2023; Kaiser et al., 2022, 2019; Peterson et al., 2025). Nevertheless, the predictive value of the presented findings is still a matter of speculation, since the currently investigated sample only included healthy and young individuals in a cross-sectional manner.

Contrary to our hypothesis, individuals exhibiting higher levels of neuroticism showed greater negative associations between trait RNT and both the occurrence and persistence of the canonical DMN CAP, compared to less neurotic individuals. With appropriate caution, we speculate that spending more time in a canonical DMN CAP might reflect a neural correlate of resilience to RNT in individuals at elevated risk for psychopathology. This finding is unexpected given prior evidence indicating positive associations between DMN occurrence and RNT-related processes, although considerable inconsistencies exist across studies (Belleau et al., 2022; Kaiser et al., 2019; C. Liu et al., 2023; Peterson et al., 2025; Piguet et al., 2021). One possible explanation for these discrepancies lies in methodological differences between studies. Whereas earlier studies have focused on simple associations between RNT facets and DMN CAP dynamics in depressed samples (Belleau et al., 2022, 2023; Kaiser et al., 2022, 2019; Peterson et al., 2025), the current study is– to our knowledge – the first to investigate the interaction of two major risk factors for psychopathology in healthy subjects. As such, our findings may capture neural correlates of RNT that precede the onset of mental disorders rather than reflect mechanisms associated with manifest psychopathology. Exploratory analysis of the SAL CAP revealed a similar pattern. A more pronounced negative association between trait RNT and the SAL CAP was found in neurotic individuals, compared to less neurotic individuals. With appropriate caution, we speculate that the temporal persistence of the SAL may constitute a correlate of decreased vulnerability for RNT among highly neurotic individuals. Herein, higher levels of SAL recruitment might reflect an individual’s capacity to effectively coordinate network recruitment during negative self-referential processing. This interpretation is supported by prior evidence linking decreased SAL recruitment, flexibility, and occurrence to heightened RNT and depression (Han et al., 2020; Kaiser et al., 2018, 2016; Lydon-Staley et al., 2019; Pang et al., 2018; Piguet et al., 2021; Wei et al., 2017). However, it should be noted that the neuroticism by RNT interactions is characterized by small effect sizes and only pertain to healthy individuals. Furthermore, the predictive value of our finding is a matter of speculation, due to the cross-sectional nature of the data. Accordingly, caution is advised when interpreting our findings, and additional investigations are needed to determine whether they translate to clinical populations.

Interestingly, the dynamics of the SAL, DMN, and hybrid FPN+DMN CAPs were associated with each other in accordance with their relationship to RNT and neuroticism. The rather canonical SAL and DMN CAPs were positively related to each other, so individuals who spent more time in the SAL CAP tended to spend more time in the canonical DMN state as well. Conversely, individuals who spent less time in the SAL or DMN spent more time in the hybrid FPN+DMN configuration. Together with the previously presented findings of the distinct associations of trait RNT with the SAL, DMN, and FPN+DMN CAPs, we propose the following preliminary hypothesis: Individuals prone to RNT (and neuroticism) may exhibit a broad shift away from canonical toward loosely segregated DMN configurations. We therefore cautiously speculate that the dissolved segregation of functionally antagonistic networks over time – especially segregations contrasting the DMN from other networks implicated in attentional or cognitive control – might be crucial to the dynamic neural underpinnings of RNT. Our hypothesis is informed by prior studies reporting associations between RNT facets and the expression of hybrid network configurations in depressed samples (Belleau et al., 2022, 2023; Kaiser et al., 2022, 2019; Peterson et al., 2025). However, to our knowledge, no studies have directly examined a generalized shift from canonical to hybrid network configurations in depression or RNT. Consequently, the present interpretation remains preliminary and should be regarded as hypothesis-generating rather than confirmatory.

Several limitations raise unresolved questions. First, our sample was composed of relatively young and educated individuals, with subjects excluded if they had been diagnosed with or been in treatment for a mental disorder in the 12 months prior to the fMRI scan. Consequently, further research is needed to support the translation of our results to clinical samples or the broader population. Another aspect to consider is that order effects could have contaminated the control condition during the RWIT. Although the rumination and worry conditions were balanced across subjects, the distraction condition was always presented last, which may have caused fatigue, spillover, or simple order effects to contaminate the control condition with increased mind-wandering and consequently interfered with task engagement. Additionally, the semi-fixed order does not allow causal conclusions regarding the effect of experimental induction of RNT on brain measures. Future studies should take these caveats into account, by fully randomizing the order of the conditions. Additionally, the cross-sectional design limits our ability to validate the predictive value of our findings and causal relationships. A notable strength of our study is the rigorous inclusion of covariates of no interest, ruling out the possibility that associations with RNT are driven by general vulnerability for internalizing psychopathology, age or sex (Ormel et al., 2013, 2004). Despite efforts to improve effect sizes and reliability, only the association between trait RNT and counts of the anterior DMN CAP, as well as the hybrid FPN+DMN CAP, survived rigorous FDR correction, underscoring the need for replication in larger samples (DeYoung et al., 2025).

## Conclusion

To the best of our knowledge, this is the first study to examine the interaction between RNT and neuroticism in the context of large-scale network dynamics during negative self-referential processing. Our results extend previous findings by showing that RNT is associated with broad abnormalities in all networks that are part of the triple network model proposed by Menon et al. (Menon, 2011, 2023). The insights presented here may prove useful for the development of neurobiologically informed theories of RNT. However, further research is required to reach definitive conclusions.

## Supporting information

10.1017/S0033291726103572.sm001 Meiering et al. supplementary material Meiering et al. supplementary material

## Supplementary material

The supplementary material for this article can be found at http://doi.org/10.1017/S0033291726103572.
