# Dynamic coactivation patterns during repetitive negative thinking: A cross-sectional fMRI study – ERRATUM

**DOI:** 10.1017/S0033291726105145

**Published:** 2026-07-30

**Authors:** Marvin Sören Meiering, Emily Belleau, David Weigner, Rebecca Gruzman, Diego Pizzagalli, Sören Enge, Simone Grimm

When this article was published in Psychological Medicine it omitted to highlight that the authors Sören Enge and Simone Grimm contributed equally to the manuscript. The article has been updated to reflect this.

The Publisher apologises for this omission.
