# Supplementary material for: Dynamic coactivation patterns during repetitive negative thinking: A cross-sectional fMRI study
Source: Psychol Med. 2026 Mar 5;56:e67. doi: 10.1017/S0033291726103572 (PMC12969223; doi:10.1017/S0033291726103572)
Supplement: Meiering et al. supplementary material [file S0033291726103572sup001.pdf]

Supplement

to

Dynamic Coactivation Patterns during Repetitive Negative Thinking: A Cross-Sectional fMRI study

Marvin S. Meiering[1,2], Emily L. Belleau[3,4], David Weigner[1,2], Rebecca Gruzman[1,2], Diego A. Pizzagalli[3,4], Sören Enge[1,†], & Simone Grimm[1,5,†]

[1]Institute of Neuroscience and Biopsychology for Clinical Application, MSB Medical School Berlin, Rüdesheimer Straße 50, 14197, Berlin, Germany

[2]Department of Education and Psychology, Freie Universität Berlin, Habelschwerdter Allee 45, 14195, Berlin, Germany

[3]Center for Depression, Anxiety and Stress Research, McLean Hospital, Belmont, MA, USA

[4]Department of Psychiatry, Harvard Medical School, Boston, MA, USA

[5]Department of Psychiatry, Psychotherapy and Psychosomatics, Psychiatric University Hospital Zurich, University of Zurich, Zurich, Switzerland

† shared last authorship

Corresponding author:

Marvin S. Meiering, M.Sc.

Institute of Neuroscience and Biopsychology for Clinical Application

MSB Medical School Berlin

Rüdesheimer Straße 50

14197 Berlin

E-Mail: marvin.meiering@medicalschool-berlin.de

Rumination and Worry Induction Task (RWIT): Stimuli

RUMINATION

In this session, you will be shown 4 sentences one after the other for 90 seconds each. Use the sentences to think carefully about the causes of the negative event. Think about this for the entire duration of the measurement and please keep your eyes open.

- Think: Which of my personality traits cause me to react so negatively in such moments?
- Think: What does the negative memory say about me as a person? What are the similarities and differences compared to others?
- Think: Why do these events happen to me and don't happen to others?
- Think: Why can't I handle events like this better?

WORRY

In this exercise, you will be shown 4 sentences one after the other for 90 seconds each. Use the sentences to think carefully about the possible consequences of the negative event. Think about this for the entire duration of the measurement and please keep your eyes open.

- Think: How could the event have a negative impact on my future?
- Think: What could cause the event to happen to me again?
- Think: How could the event negatively affect my relationships with other people?
- Think: How could the event negatively affect my current and future well-being?

DISTRACTION

In this session, you will be shown 4 sentences one after the other for 90 seconds each. Focus your attention on the sentences and imagine their content as vividly and detailed as possible. Imagine the respective content for the entire duration of the measurement and please keep your eyes open.

- Imagine: A typical classroom from school.
- Imagine: Raindrops running down a window.
- Imagine: Clouds forming in the sky.
- Imagine: A train stopping at a station.

Latent Variable modeling

The results of the confirmatory factor analysis are under consideration for publication elsewhere and were published as a preprint (Meiering, Weigner, Gruzman, Enge, & Grimm, 2025). The following information are a citation of Meiering et al. (2025):

In line with recent suggestions to improve effect sizes for brain-behavior relationships by increasing the reliability of self-report questionnaires (DeYoung et al., 2025), a confirmatory factor analysis was used to estimate the expression of RNT as a latent factor indicated by the RSQ brooding subscale, CERQ rumination subscale, PSWQ and PTQ. First, indicators were z-standardized to ensure similar scaling of the observed variables. To test for multivariate normality, the energy and Mardia tests were used as implemented in the MVN package (Korkmaz, Goksuluk, & Zararsiz, 2014). Subsequently, the R package lavaan was employed to fit the model and extract model fit indices, factor loadings and their associated 95% bootstrap confidence intervals as well as RNT factor scores on the single subject level (Rosseel, 2012). A maximum likelihood estimator was used to fit the model, assuming CFI ≥ .95, TLI ≥ .9 and SRMR ≤ .08 to indicate good fit (Byrne, 1994; Hu & Bentler, 1999). RMSEA will be reported for descriptive reasons but not interpreted due to its known unreliability in face of small degree of freedoms and small sample sizes (Kenny, Kaniskan, & McCoach, 2015). The same analytical regimen was performed to extract neuroticism factor scores based on the Negative Affectivity dimension of the ATQ, the Negative Emotionality dimension of the BFI-2, the Neuroticism scale of the BFI-K, the Negative Affect scale of the PANAS (trait) as well as the trait anxiety scale of the STAI.

Repetitive Negative Thinking

A latent factor model was fitted using confirmatory factor analysis including questionnaire data from 122 subjects. Energy and Mardia tests suggested violation of the multivariate normality assumption (Energy: *E-statistic* = 1.342, p = .016; Mardia: $skewness_{statistic}$ = 60.070, $skewness_{p\text{-}value}$ = .005, $kurtosis_{statistic}$ =-0.420, $kurtosis_{p\text{-}value}$ = .675). The measurement model included four indicators (PTQ, RSQ brooding subscale, CERQ rumination subscale, PSWQ) and a single higher-order factor (RNT). Model fit indices provided consistent evidence for excellent model fit ($\chi 2(1)$ = 1.246, p_{boot} = .531, *CFI* = .998, *TLI* = .991, *SRMR* = .017, *RMSEA* = 0.045) and high factor loadings were observed (see table S2).

Neuroticism

A latent factor model for neuroticism was fitted. The Energy and Mardia tests suggested violation of the multivariate normality assumption (Energy: *E-statistic* = 1.750, p < .001; Mardia: $skewness_{statistic}$ = 155.606, $skewness_{p\text{-}value}$ < .001, $kurtosis_{statistic}$ = 5.377, $kurtosis_{p\text{-}value}$ < .001). The measurement model included five indicators (ATQ Negative Affectivity, BFI-2 Negative Emotionality, BFI-K Neuroticism, PANAS Negative Affect, STAI trait Anxiety) and a single higher-order factor (Neuroticism). Model fit indices provided evidence for

excellent model fit ($\chi2(4)$ = 9.659, p_{boot} = .180, *CFI* = .985, *TLI* = .963, *SRMR* = .032, *RMSEA* = 0.108) and sufficiently high factor loadings were observed (see Table S2).

Table S1. Descriptive statistics of questionnaires included in the CFA

Questionnaire	*M*	*SD*
RSQ – Brooding	9.33	3.21
PSWQ	43.55	11.00
CERQ- Rumination	12.01	3.23
PTQ – Total	37.08	9.22
PANAS trait- Negative Affect	1.52	0.58
ATQ – Negative Affectivity	94.07	20.00
BFI-2 – Negative Emotionality	25.59	7.16
BFI-2 short version - Neuroticism	10.68	3.48
STAI – Trait Anxiety	64.4	21.47

Notes. RSQ = Response Styles Questionnaire, PSWQ = Penn State Worry Questionnaire, CERQ = Cognitive Emotion Regulation Questionnaire, PTQ = Perseverative Thinking Questionnaire, PANAS = Positive And Negative Affect Schedule, ATQ = Adult Temperament Questionnaire, BFI = Big Five Inventory, STAI = State Trait Anxiety Inventory.

Table S2. CFA factor loadings

Scale	*Unstandardized factor loading*	λ	*95%-BCI*	*z*
	Repetitive Negative Thinking			
PTQ	0.809	.832	[0.680, 0.948]	11.952
RSQ Brooding	0.678	.698	[0.524, 0.856]	8.011
PSWQ	0.754	.776	[0.632, 0.901]	10.970
CERQ Rumination	0.537	.552	[0.387, 0.710]	6.480
	Neuroticism			
PANAS Negative Affect (trait)	0.679	.511	[0.352, 0.991]	4.103
ATQ Negative Affectivity	1.008	.758	[0.848, 1.177]	11.870
BFI-2 Negative Emotionality	1.225	.921	[1.124, 1.335]	22.652
STAI Anxiety (trait)	1.094	.823	[0.988, 1.223]	18.428
BFI-K Negative Emotionality	1.195	.899	[1.085, 1.299]	22.132

Note: 95%-BCI reflect bootstrap confidence intervals for the unstandardized factor loadings. λ = standardized factor loadings, PTQ = Perseverative Thinking Questionnaire, RSQ = Response Styles Questionnaire, PSWQ = Penn State Worry Questionnaire, CERQ = Cognitive Emotion Regulation Questionnaire, PANAS = Positive And Negative Affect Schedule, ATQ = Adult Temperament Questionnaire, BFI-2 = Big Five Inventory, STAI = State Trait Anxiety Inventory, BFI-K = Big Five Inventory Short Version, BFI-2 = Big Five Inventory 2.

MRI image acquisition and analysis

MRI Acquisition. Brain images were acquired using a 3 Tesla MRI scanner (PRISMA fit, Siemens Medical Systems, Erlangen, Germany). The anatomical images were acquired by means of a 3D T1 weighted sequence (Magnetization Prepared Rapid Acquisition Gradient Echo sequence, TR=2.3 s, TE=3.03 ms, slices = 192, voxel size = 1x1x1 mm, flip angle = 9°, FOV = 256x256x192 mm). Functional brain images were acquired using a T2* weighted gradient echo-planar imaging sequence sensitive to the BOLD effect (Blood Oxygen Level Dependent), accelerated by a factor of 3 (TR = 1 s, TE = 0.3 ms, slices = 39, voxel size = 3x3x3 mm, flip angle = 65°, matrix = 64x64, FOV = 192x192x140 mm, MB factor = 3). Moreover, to improve registration a fieldmap was obtained employing a double-echo gradient echo field map sequence (TR = 468 ms, TE = 4.92 / 7.38 ms, slices = 39, voxel size = 3x3x3 mm, flip angle = 60°, matrix = 64x64, FOV = 192x192x140).

Brain image preprocessing and analysis were carried out using FEAT (FMRI Expert Analysis Tool; (Woolrich, Behrens, Beckmann, Jenkinson, & Smith, 2004; Woolrich, Behrens, & Smith, 2004; Woolrich et al., 2009; Woolrich, Ripley, Brady, & Smith, 2001)) version 6, as part of FSL (FMRIB's Software Library; (Jenkinson, Beckmann, Behrens, Woolrich, & Smith, 2012; Smith et al., 2004; Woolrich et al., 2009)). T1 anatomical data were biasfield corrected and aligned to the MNI152 standard space using linear alignment via FSL FLIRT with 12 degrees of freedom and subsequently refined non-linearly as implemented in FSL FNIRT. The processing of the functional brain images included realignment of participants' head motion, correction for EPI distortions using fieldmap data, and a 5-mm FWHM spatial smoothing. To identify and correct for more subtle effects of head motion FSL's MELODIC (Beckmann & Smith, 2004, 2005) was used to extract independent data components followed by ICA-AROMA (Pruim, Mennes, Buitelaar, & Beckmann, 2015; Pruim, Mennes, Van Rooij, et al., 2015) to identify and remove secondary effects of head motion. Finally, a temporal 0.01 Hz high-pass filter was applied to remove scanner drifts. Furthermore, a transformation from the functional space to the T1 anatomical space using FSL Boundary Based Registration was obtained. Eventually, the transformation was combined with the T1 to MNI152 registration to transfer the functional data from the individual's native space to the MNI152 standard space. After applying a high-pass filter to account for scanner drift, the blocks representing the three conditions of interest (rumination, worry and distraction) were extracted from the fMRI time series.

Figure S1. Results consensus clustering

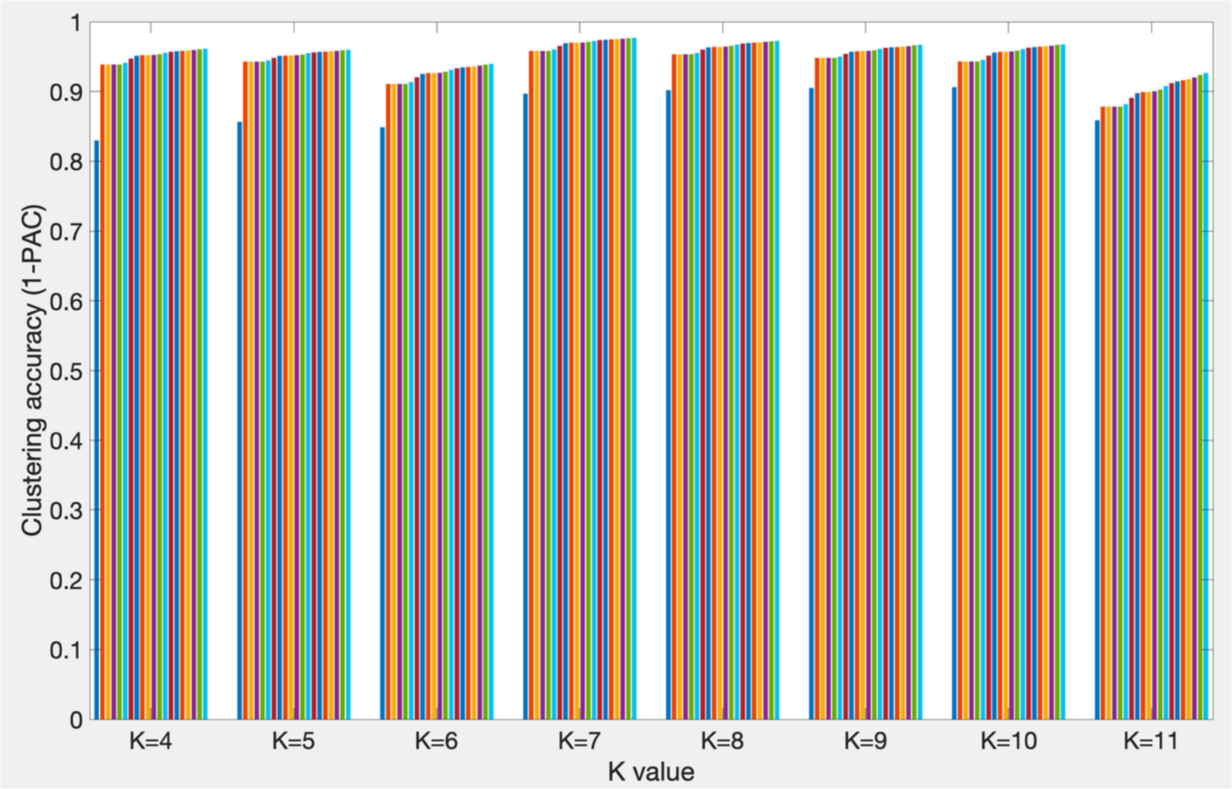

Notes. PAC = percentage of ambiguously clustered pairs. On the y axis, higher values indicate better clustering accuracy.

Figure S2. Results consensus clustering

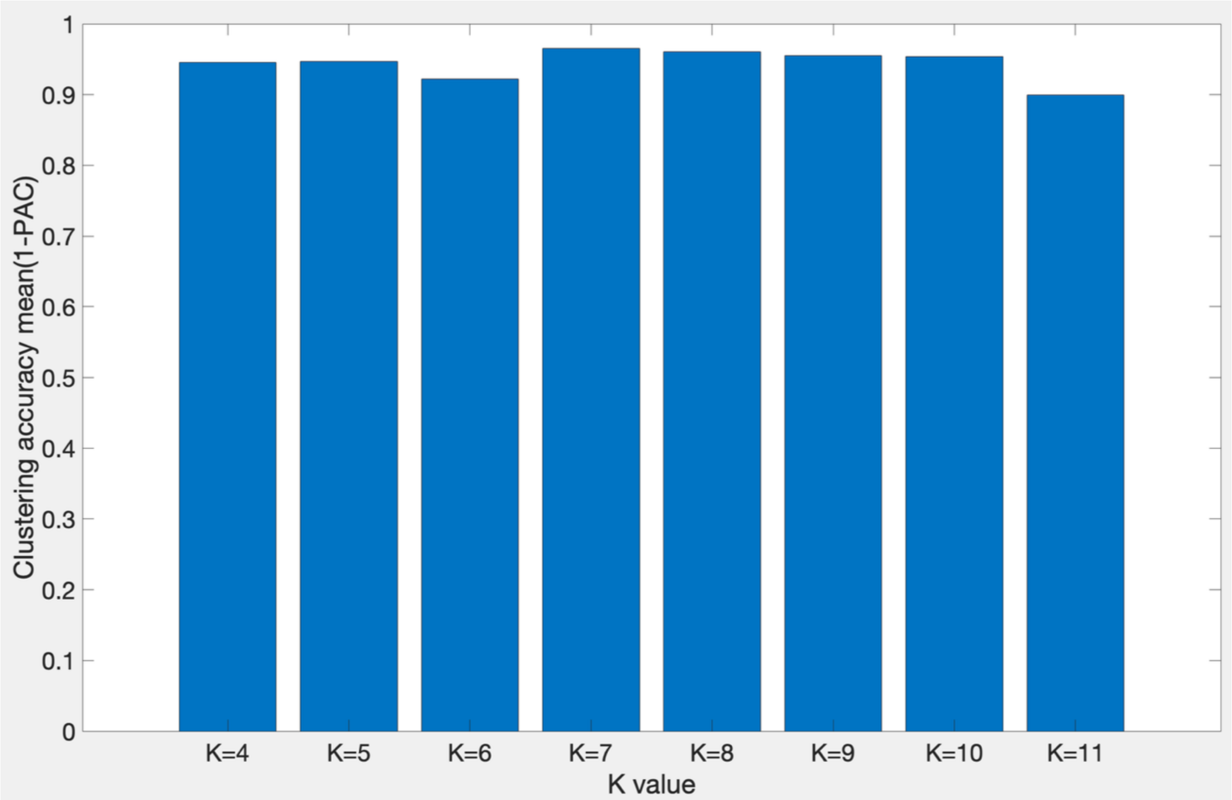

Notes. PAC = percentage of ambiguously clustered pairs. On the y axis, higher values indicate better clustering accuracy.

Figure S3. CAP6 reflecting noise

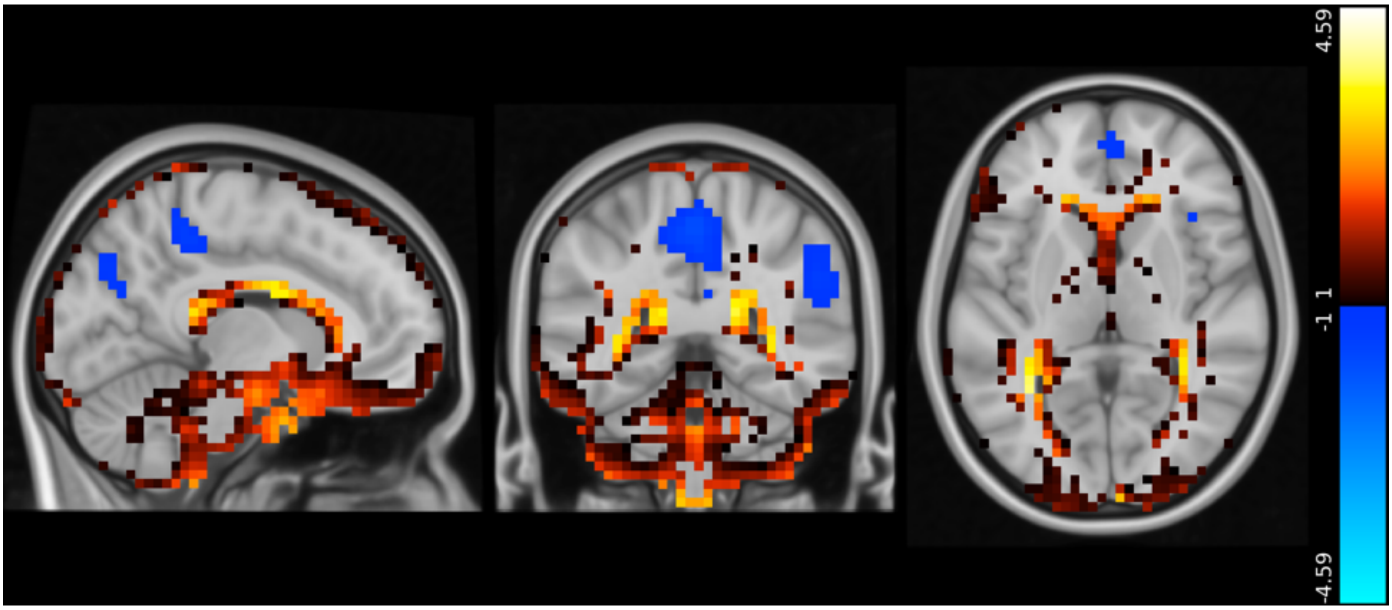

Note. Color bar indicates z-values that reflect the contribution of the regions to CAP6. Most of the contributing voxels are located at the edges to the ventricles, brain boundaries and white matter.

Figure S4. CAP characterization

MAPS | *SPATIAL CROSS CORRELATION* | *REGIONS*

CAP1

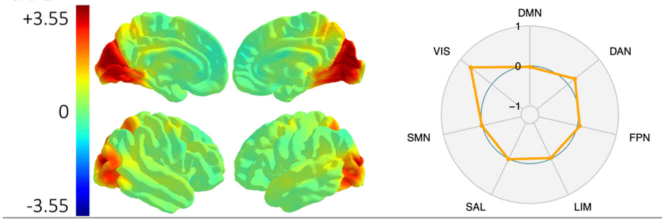

Activation: occipital cortex, cerebellum
Deactivation:-

CAP2

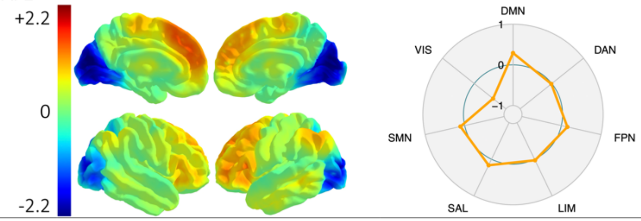

Activation: SFG, mPFC, ACC
Deactivation: occipital cortex, precuneous, cerebellum, RSC

CAP3

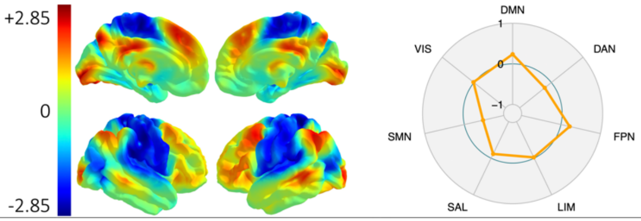

Activation: PCC, precuneous, SFG, PPC, lPFC, AI, MFG
Deactivation: PI, postcentral gyrus, precentral gyrus

CAP4

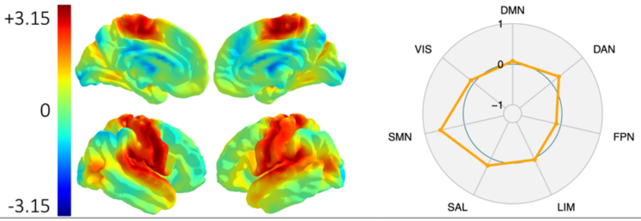

Activation: planum temporale, postcentral gyrus, precentral gyrus
Deactivation: -

CAP5

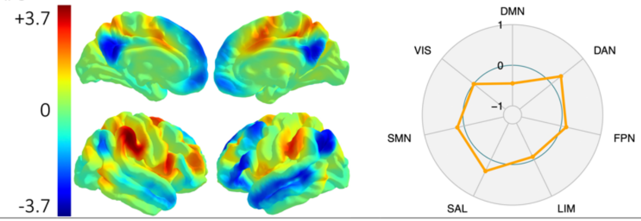

Activation: AI, SMG, inferior parietal sulcus, mCC, FP
Deactivation: PCC, precuneous, mPFC, IFG, TP, lPFC, PPC

CAP6

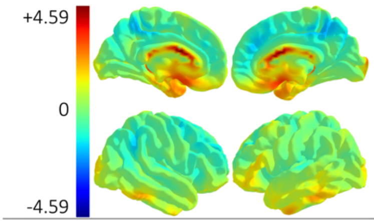

Artifacts/noise

CAP7

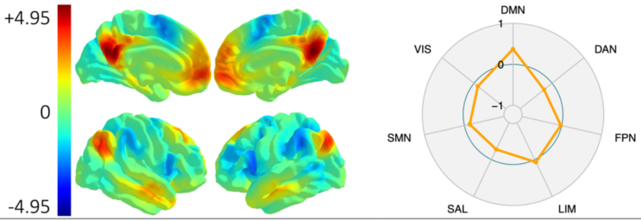

Activation: mPFC, AG, PCC, precuneous, hippocampus
Deactivation: AI, FP

Notes. SFG = superior frontal gyrus, mPFC = medial prefrontal cortex, ACC = anterior cingulate cortex, RSC = retrosplenial cortex, PCC = posterior cingulate cortex, PPC = posterior parietal cortex, lPFC = lateral prefrontal cortex, AI = anterior insula, MFG = middle frontal gyrus, PI = posterior insula, SMG = superior marginal gyrus, mCC = midcingulate cortex, FP = frontal pole, IFG = inferior frontal gyrus, TP = temporal pole, AG = angular gyrus, VIS = visual network, DMN = default mode network, FPN = frontoparietal network, SMN = sensorymotor network, SAL = salience network / ventral attention network, DAN = dorsal attention network, LIM = limbic network. Color bars depict local z-values.

Table S3. Spatial cross-correlations between CAPs and Yeo's 7 network atlas.

CAP	DMN	DAN	FPN	LIM	VAN	SMN	VN
1	-0.024	0.221	0.061	-0.020	0.013	0.012	0.678
2	0.291	-0.015	0.152	0.028	0.164	0.108	-0.593
3	0.236	-0.209	0.221	-0.022	-0.116	-0.473	0.014
4	0.077	0.244	-0.114	0.038	0.199	0.609	0.099
5	-0.447	0.302	0.132	-0.077	0.315	0.171	-0.002
6	0.008	-0.082	-0.144	0.299	-0.097	-0.091	0.088
7	0.373	-0.251	-0.023	0.070	-0.274	-0.135	-0.120

Notes. CAP = coactivation pattern, DMN = default mode network, DAN = dorsal attention network, FPN = frontoparietal network, LIM = limbic network, VAN = ventral attention network, SMN = somatomotor network, VN = visual network.

Task engagement

Results

Within-subject differences between task conditions (RUM, WOR, RNT, DIS) were tested for differences using nonparametric 95% bootstrap confidence intervals (95%-BCI) with 10000 iterations. Bootstrap confidence intervals of differences between conditions of interest (RNT>DIS and WOR>RUM) revealed increased counts and persistence of CAP5 (SAL) during RNT compared to distraction, but no differences between worry and rumination (see Table S4, Table S5 and Figure S5). Also, CAP7 (DMN) was expressed less often during RNT compared to distraction and more often during worry compared to rumination. Furthermore, greater persistence in CAP7 (DMN) was observed during worry compared to rumination.

Table S4. Descriptive statistics

	RUM			*WOR*			*RNT*			*DIS*		
	M	*SD*	*95% BCI*	*M*	*SD*	*95% BCI*	*M*	*SD*	*95% BCI*	*M*	*SD*	*95% BCI*
Count												
CAP2	15.7	4.2	[14.9, 16.5]	15.8	5.5	[14.8, 16.8]	15.8	3.9	[15.0, 16.5]	15.1	5.14	[14.1, 16.1]
CAP3	18.1	17.1	[16.2, 19.9]	18.6	18.3	[16.7, 20.5]	18.4	16.9	[16.6, 20.2]	19.2	18.8	[17.2, 21.1]
CAP5	17.1	15.4	[15.6, 18.5]	17.2	15.9	[15.7, 18.7]	17.1	15.2	[15.7, 18.6]	14.6	13.4	[13.3, 15.8]
CAP7	15.0	13.8	[13.5, 16.4]	16.4	15.0	[14.8, 18.0]	15.7	13.7	[14.2, 17.2]	16.7	16.3	[14.9, 18.4]
Persistence												
CAP2	39.0	14.8	[36.2, 41.7]	39.7	17.3	[36.4, 42.8]	39.3	12.8	[37.0, 41.7]	38.6	16.8	[35.5, 41.6]
CAP3	18.1	17.1	[16.3, 19.9]	18.6	18.3	[16.6, 20.6]	18.4	16.9	[16.6, 20.2]	19.2	18.8	[17.1, 21.1]
CAP5	17.1	15.4	[15.6, 18.5]	17.2	15.9	[15.7, 18.5]	17.1	15.2	[15.7, 18.5]	14.6	13.4	[13.3, 15.8]
CAP7	15.0	13.8	[13.5, 16.5]	16.4	15.0	[14.8, 18.0]	15.7	13.7	[14.2, 17.2]	16.7	16.3	[14.9, 18.4[

Notes. CAP = coactivation pattern, RUM = rumination, WOR = worry, RNT = repetitive negative thinking, DIS = distraction, BCI = bootstrap confidence intervals.

Table S5. Task engagement.

CAP	*p-value*	RNT>DIS		WOR>RUM	
		MD	*95% BCI*	*MD*	*95% BCI*
Count					
CAP2	.384	0.646	[-0.283, 1.580]	-0.129	[-0.925, 1.170]
CAP3	.366	-0.795	[-1.730, 0.174]	0.508	[-0.614, 1.630]
CAP5	<.001	2.590	[1.860, 3.320]	0.116	[-0.613, 0.844]
CAP7	.096	-1.010	[-1.980, -0.044]	1.370	[0.420, 2.300]
Persistence					
CAP2	.916	0.795	[-2.460, 4.150]	0.643	[-2.930, 4.250]
CAP3	.354	-0.795	[-1.730, 0.169]	0.508	[-0.621, 1.650]
CAP5	<.001	2.59	[1.850, 3.300]	0.116	[-0.622, 0.843]
CAP7	.016	-1.010	[-1.980, -0.032]	1.370	[0.402, 2.310]

Notes. CAP = coactivation pattern; DIS = distraction, RUM = rumination, WOR = worry, RNT = repetitive negative thinking, BCI = bootstrap confidence intervals.

Figure S5. Differences between task conditions

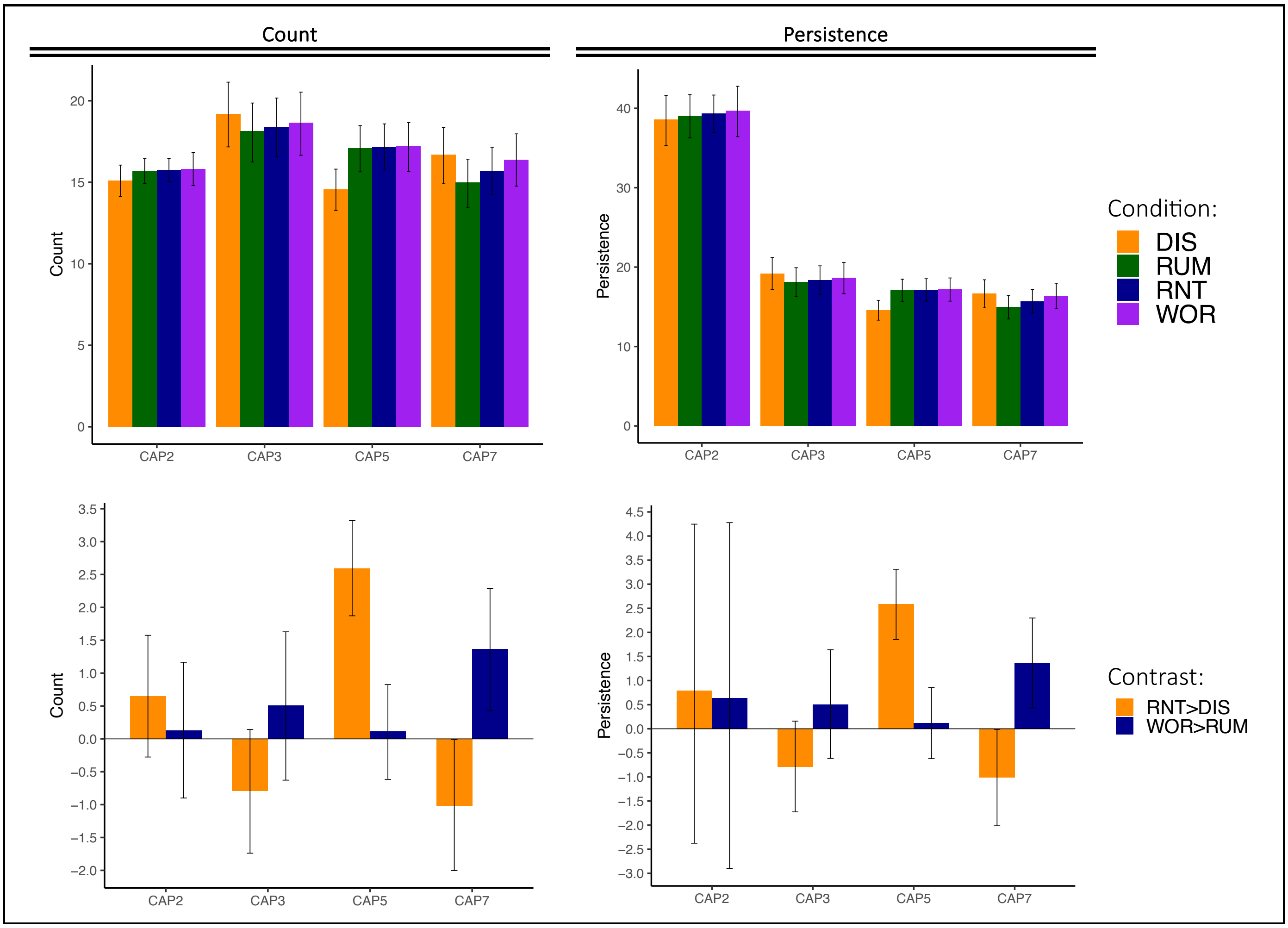

Notes. DIS = distraction, RUM = rumination, RNT = repetitive negative thinking, WOR = worry. Error bars reflect 95% bootstrap confidence intervals. Plots at the top represent raw values, plots at the bottom reflect differences between conditions.

Discussion

Substantiating a previous report (Piguet, Karahanoğlu, Saccaro, Van De Ville, & Vuilleumier, 2021), increased counts and persistence of a SAL CAP was observed during RNT compared to DIS. Consistent with this notion, previous research has suggested that individuals with low SAL flexibility and high negative mood tend to report more severe RNT (Lydon-Staley et al., 2019). The SAL is considered to be critically involved in detecting salient stimuli, shifting of attentional resources and subsequently orchestrating large-scale network recruitment to deal with environmental demands (Menon, 2011, 2023). Accordingly, sufficient flexibility is of outmost importance to the SAL. A rigidly acting SAL may hinder or decelerate network changes that are necessary to reallocate attentional resources from problem- to solution-oriented thinking modes. A different interpretational route highlights the time-dependent expression of the SAL as a compensatory mechanism. More time and therefore more neural resources are needed to attain necessary network shifts during RNT.

Furthermore, reduced counts and persistence of a canonical DMN CAP emerged during state RNT compared to distraction. No consensus has been reached whether RNT and DMN counts are associated (Belleau et al., 2022; Kaiser et al., 2019; Katayama et al., 2024; Liu et al., 2023; Piguet et al., 2021). However, a recent study provided evidence that spending more time in a canonical DMN CAP may actually serve as a marker of treatment response in individuals with depression (Kaiser et al., 2022). In this context, state RNT may be associated with less persistence and time spent in the canonical DMN, suggesting an impaired ability to sustain the recruitment of clearly segregated canonical networks. Notably, the unstandardized effect sizes for the differences between conditions are small. During RNT, participants spent on average an additional two to three seconds in the SAL CAP and about one second less in the DMN CAP, compared to the distraction condition. Given the overall duration of each condition (360 seconds), these differences are considerably small, and further studies will be needed to evaluate the potential clinical utility of this marker.

Table S6. Regression results.

CAP	*B*	*95%-CI (B)*	*β*	*95%-CI (β)*	*p*	*p-FDR*
Self-report: trait RNT*neuroticism interaction						
			Count			
Contrast: RNT>DIS						
CAP2	0.960	[-0.497, 2.182]	0.114	[-0.060, 0.259]	.179	.239
CAP3	-0.026	[-2.221, 1.860]	-0.003	[-0.294, 0.246]	.944	.944
CAP5	-0.739	[-1.929, 0.505]	-0.099	[-0.263, 0.076]	.222	.332
CAP7	-0.438	[-2.060, 0.820]	-0.053	[-0.256, 0.110]	.475	.723
Contrast: RNT						
CAP2	0.022	[-1.593, 1.267]	0.003	[-0.230, 0.175]	.999	.999
CAP3	1.434	[-0.605, 3.482]	0.176	[-0.072, 0.432]	.162	.324
CAP5	-2.030	[-3.694,-0.044]	-0.261	[-0.475,-0.013]	.047	.188
CAP7	-1.562	[-2.974,-0.173]	-0.217	[-0.414,-0.024]	.032	.164
			Persistence			
Contrast: RNT>DIS						
CAP2	4.248	[-0.718, 8.841]	0.146	[-0.031, 0.304]	.087	.184
CAP3	0.760	[-7.966, 7.533]	0.029	[-0.301, 0.291]	.898	.944
CAP5	-2.323	[-6.085, 1.322]	-0.097	[-0.259, 0.059]	.191	.332
CAP7	-1.133	[-6.380, 2.953]	-0.042	[-0.237, 0.116]	.542	.723
Contrast: RNT						
CAP2	1.878	[-3.246, 5.759]	0.081	[-0.140, 0.248]	.409	.467
CAP3	5.558	[-1.553, 12.885]	0.198	[-0.053, 0.467]	.121	.323
CAP5	-6.162	[-11.152,-0.640]	-0.255	[-0.457,-0.031]	.034	.188
CAP7	-4.134	[-8.175,-0.226]	-0.191	[-0.378,-0.008]	.041	.164
Self-report: trait RNT factor scores						
			Count			
Contrast: RNT>DIS						
CAP2	2.465	[0.899, 4.045]	0.430	[0.153, 0.716]	.004	.032
CAP3	-0.664	[-2.589, 1.127]	-0.127	[-0.493, 0.223]	.432	.691
CAP5	-1.167	[-2.904, 0.556]	-0.231	[-0.575, 0.113]	.179	.332
CAP7	1.278	[-0.555, 3.167]	0.228	[-0.099, 0.565]	.188	.394
Contrast: RNT						
CAP2	-1.233	[-2.933, 0.198]	-0.259	[-0.611, 0.043]	.092	.184
CAP3	2.468	[0.522, 4.872]	0.447	[0.085, 0.881]	.014	.056
CAP5	-0.924	[-2.888, 0.948]	-0.176	[-0.555, 0.189]	.303	.346
CAP7	-0.062	[-1.662, 1.744]	-0.013	[-0.345, 0.357]	.890	.991
			Persistence			
Contrast: RNT>DIS						
CAP2	6.927	[0.589, 13.293]	0.351	[0.023, 0.673]	.033	.132
CAP3	-1.168	[-7.864, 5.263]	-0.065	[-0.428, 0.295]	.707	.943
CAP5	-3.280	[-9.126, 2.389]	-0.202	[-0.566, 0.148]	.249	.332
CAP7	4.156	[-1.948, 10.452]	0.227	[-0.101, 0.567]	.197	.394
Contrast: RNT						
CAP2	-3.503	[-8.892, 1.115]	-0.225	[-0.565, 0.075]	.137	.219
CAP3	9.233	[2.120, 17.580]	0.486	[0.106, 0.914]	.012	.056
CAP5	-1.651	[-7.889, 3.975]	-0.101	[-0.495, 0.263]	.524	.524
CAP7	0.260	[-4.556, 5.664]	0.018	[-0.313, 0.373]	.991	.991

Notes. B = unstandardized coefficient, β = standardized coefficient, BCI = bootstrap confidence intervals, DIS = distraction, RNT = repetitive negative thinking. All results are corrected for the effects of age and sex.

References

Beckmann, C. F., & Smith, S. M. (2004). Probabilistic Independent Component Analysis for Functional Magnetic Resonance Imaging. *IEEE Transactions on Medical Imaging*, *23*(2), 137–152. doi: 10.1109/TMI.2003.822821

Beckmann, C. F., & Smith, S. M. (2005). Tensorial extensions of independent component analysis for multisubject FMRI analysis. *NeuroImage*, *25*(1), 294–311. doi: 10.1016/j.neuroimage.2004.10.043

Belleau, E. L., Bolton, T. A. W., Kaiser, R. H., Clegg, R., Cárdenas, E., Goer, F., … Pizzagalli, D. A. (2022). Resting state brain dynamics: Associations with childhood sexual abuse and major depressive disorder. *NeuroImage: Clinical*, *36*, 103164. doi: 10.1016/j.nicl.2022.103164

Byrne, B. M. (1994). *Structural equation modeling with EQS and EQS/Windows: Basic concepts, applications, and programming* (7. print). Thousand Oaks, Calif.: Sage.

DeYoung, C. G., Hilger, K., Hanson, J. L., Abend, R., Allen, T. A., Beaty, R. E., … Wacker, J. (2025). Beyond Increasing Sample Sizes: Optimizing Effect Sizes in Neuroimaging Research on Individual Differences. *Journal of Cognitive Neuroscience*, 1–12. doi: 10.1162/jocn_a_02297

Hu, L., & Bentler, P. M. (1999). Cutoff criteria for fit indexes in covariance structure analysis: Conventional criteria versus new alternatives. *Structural Equation Modeling: A Multidisciplinary Journal*, *6*(1), 1–55. doi: 10.1080/10705519909540118

Jenkinson, M., Beckmann, C. F., Behrens, T. E. J., Woolrich, M. W., & Smith, S. M. (2012). FSL. *NeuroImage*, *62*(2), 782–790. doi: 10.1016/j.neuroimage.2011.09.015

Kaiser, R. H., Chase, H. W., Phillips, M. L., Deckersbach, T., Parsey, R. V., Fava, M., … Pizzagalli, D. A. (2022). Dynamic Resting-State Network Biomarkers of Antidepressant Treatment Response. *Biological Psychiatry*, S0006322322011544. doi: 10.1016/j.biopsych.2022.03.020

Kaiser, R. H., Kang, M. S., Lew, Y., Van Der Feen, J., Aguirre, B., Clegg, R., … Pizzagalli, D. A. (2019). Abnormal frontoinsular-default network dynamics in adolescent depression and rumination: A preliminary resting-state co-activation pattern analysis. *Neuropsychopharmacology*, *44*(9), 1604–1612. doi: 10.1038/s41386-019-0399-3

Katayama, N., Shinagawa, K., Hirano, J., Kobayashi, Y., Nakagawa, A., Umeda, S., … Mimura, M. (2024, October 21). *Dynamic Neural Network Modulation Associated with Rumination in Major Depressive Disorder: A Prospective Observational Comparative Analysis of Cognitive Behavioral Therapy and Pharmacotherapy*. ResearchSquare. doi: 10.21203/rs.3.rs-4806538/v1

Kenny, D. A., Kaniskan, B., & McCoach, D. B. (2015). The Performance of RMSEA in Models With Small Degrees of Freedom. *Sociological Methods & Research*, *44*(3), 486–507. doi: 10.1177/0049124114543236

Korkmaz, S., Goksuluk, D., & Zararsiz, G. (2014). MVN: An R Package for Assessing Multivariate Normality. *The R Journal*, *6*(2), 151. doi: 10.32614/RJ-2014-031

Liu, C., Belleau, E. L., Dong, D., Sun, X., Xiong, G., Pizzagalli, D. A., … Yao, S. (2023). Trait- and state-like co-activation pattern dynamics in current and remitted major depressive disorder. *Journal of Affective Disorders*, *337*, 159–168. doi: 10.1016/j.jad.2023.05.074

Lydon-Staley, D. M., Kuehner, C., Zamoscik, V., Huffziger, S., Kirsch, P., & Bassett, D. S. (2019). Repetitive negative thinking in daily life and functional connectivity among default mode, fronto-parietal, and salience networks. *Translational Psychiatry*, *9*(1), 234. doi: 10.1038/s41398-019-0560-0

Meiering, M. S., Weigner, D., Gruzman, R., Enge, S., & Grimm, S. (2025, August 5). *An Investigation of the Interaction of Trait Repetitive Negative Thinking and Neuroticism on Default Mode Network Activity During Negative Self-Referential Processing: A Cross-Sectional fMRI Study*. Open Science Framework. doi: 10.31219/osf.io/mgdr9_v1

Menon, V. (2011). Large-scale brain networks and psychopathology: A unifying triple network model. *Trends in Cognitive Sciences*, *15*(10), 483–506. doi: 10.1016/j.tics.2011.08.003

Menon, V. (2023). 20 years of the default mode network: A review and synthesis. *Neuron*, *111*(16), 2469–2487. doi: 10.1016/j.neuron.2023.04.023

Piguet, C., Karahanoğlu, F. I., Saccaro, L. F., Van De Ville, D., & Vuilleumier, P. (2021). Mood disorders disrupt the functional dynamics, not spatial organization of brain resting state networks. *NeuroImage: Clinical*, *32*, 102833. doi: 10.1016/j.nicl.2021.102833

Pruim, R. H. R., Mennes, M., Buitelaar, J. K., & Beckmann, C. F. (2015). Evaluation of ICA-AROMA and alternative strategies for motion artifact removal in resting state fMRI. *NeuroImage*, *112*, 278–287. doi: 10.1016/j.neuroimage.2015.02.063

Pruim, R. H. R., Mennes, M., Van Rooij, D., Llera, A., Buitelaar, J. K., & Beckmann, C. F. (2015). ICA-AROMA: A robust ICA-based strategy for removing motion artifacts from fMRI data. *NeuroImage*, *112*, 267–277. doi: 10.1016/j.neuroimage.2015.02.064

Rosseel, Y. (2012). lavaan: An R Package for Structural Equation Modeling. *Journal of Statistical Software*, *48*(2), 1–36. doi: 10.18637/jss.v048.i02

Smith, S. M., Jenkinson, M., Woolrich, M. W., Beckmann, C. F., Behrens, T. E. J., Johansen-Berg, H., … Matthews, P. M. (2004). Advances in functional and structural MR image analysis and implementation as FSL. *NeuroImage*, *23*, 208–219. doi: 10.1016/j.neuroimage.2004.07.051

Woolrich, M. W., Behrens, T. E. J., Beckmann, C. F., Jenkinson, M., & Smith, S. M. (2004). Multilevel linear modelling for FMRI group analysis using Bayesian inference. *NeuroImage*, *21*(4), 1732–1747. doi: 10.1016/j.neuroimage.2003.12.023

Woolrich, M. W., Behrens, T. E. J., & Smith, S. M. (2004). Constrained linear basis sets for HRF modelling using Variational Bayes. *NeuroImage*, *21*(4), 1748–1761. doi: 10.1016/j.neuroimage.2003.12.024

Woolrich, M. W., Jbabdi, S., Patenaude, B., Chappell, M., Makni, S., Behrens, T., … Smith, S. M. (2009). Bayesian analysis of neuroimaging data in FSL. *NeuroImage*, *45*(1), S173–S186. doi: 10.1016/j.neuroimage.2008.10.055

Woolrich, M. W., Ripley, B. D., Brady, M., & Smith, S. M. (2001). Temporal Autocorrelation in Univariate Linear Modeling of FMRI Data. *NeuroImage*, *14*(6), 1370–1386. doi: 10.1006/nimg.2001.0931
